# Endothelial Cell Proteins as Biomarkers in Susac Syndrome

**DOI:** 10.1002/acn3.70508

**Published:** 2026-08-13

**Authors:** Rohit Benjamin, Yue Andy Qi, Ying Hao, Catherine DeMarino, Samuel Darko, Irene Cortese, Daniel S. Reich, María I. Gaitán, Steven Jacobson, Bridgett J. Billioux, Bryan Smith, Avindra Nath

**Affiliations:** ^1^ Section of Infections of the Nervous System National Institute of Neurological Disorders and Stroke, National Institutes of Health Bethesda Maryland USA; ^2^ Center for Alzheimer's and Related Dementias (CARD) National Institute on Aging and National Institute of Neurological Disorders and Stroke, National Institutes of Health Bethesda Maryland USA; ^3^ Experimental Immunotherapeutics Unit National Institute of Neurological Disorders and Stroke, National Institutes of Health Bethesda Maryland USA; ^4^ Translational Neuroradiology Section National Institute of Neurological Disorders and Stroke, National Institutes of Health Bethesda Maryland USA; ^5^ Viral Immunology Section National Institute of Neurological Disorders and Stroke, National Institutes of Health Bethesda Maryland USA; ^6^ Program in International Neuroinfectious Diseases National Institute of Neurological Disorders and Stroke, National Institutes of Health Bethesda Maryland USA; ^7^ Department of Neurology DC Veterans Affairs Medical Center Washington, DC USA

**Keywords:** biomarker, CNS demyelination, endothelial cell, mass spectrometry, vasculitis

## Abstract

**Objective:**

Susac syndrome (SS) is a rare CD8^+^ T cell–mediated microangiopathy affecting the brain, retina, and auditory labyrinth. Endothelial injury is thought to be a central mechanism; however, no circulating disease biomarkers are known. We performed targeted proteomic profiling to identify circulating endothelial‐associated proteins as biomarkers in SS.

**Methods:**

Serum from four cases with definite or probable SS and eight healthy controls was analyzed using data‐independent acquisition mass spectrometry. Analyses were restricted to proteins with established endothelial cell surface or membrane association, and differential abundance was assessed using detection‐based (Fisher's exact) and quantitative (probabilistic dropout analysis) modeling.

**Results:**

Six endothelial‐associated proteins demonstrated differential abundance in SS (*p* < 0.05 on unadjusted analysis). Low‐density lipoprotein receptor‐related protein 1 was increased (log_2_ fold change = +2.19, *p* = 0.0059), whereas Nectin‐2, Melanoma cell adhesion molecule, Fatty acid transport protein 4, P‐selectin, and Interleukin‐6 signal transducer were decreased (log_2_ fold change −1.38 to −1.89; all *p* < 0.05).

**Conclusions:**

Our findings suggest circulating proteins on the endothelial cell surface or membrane as potential biomarkers for SS and support endothelial dysfunction as a central disease mechanism. This pilot study provides a rationale for validation in larger, longitudinal cohorts.

**Trial Registration:**

ClinicalTrials.gov, NCT00001248 and NCT02435810

**Abbreviations**
BBB: Blood–brain barrier
CD8^+^
: Cluster of differentiation 8–positive
CSF: Cerebrospinal fluid
DIA‐MS: Data‐independent acquisition mass spectrometry
FLAIR: Fluid‐attenuated inversion recovery
IL6ST: Interleukin‐6 signal transducer
LC: Liquid chromatography
LRP1: Low‐density lipoprotein receptor–related protein 1
MCAM: Melanoma cell adhesion molecule
mRS: Modified Rankin Scale
MS: Mass spectrometry
NECTIN2: Nectin cell adhesion molecule 2
SELP: P‐selectin
SLC27A4: Solute carrier family 27 member 4
SS: Susac syndrome

## 1 Introduction

Susac syndrome (SS) is a rare autoimmune microangiopathy characterized by a clinical triad of encephalopathy, branch retinal artery occlusions, and sensorineural hearing loss [1]. Since the disease often presents with incomplete symptoms and relapses, diagnosis may be delayed, resulting in permanent neurological, visual, and auditory disability. While initially considered a small‐vessel vasculitis, SS is now known to involve cytotoxic CD8^+^ T‐cells targeting microvascular endothelial cells in principal organs [2].

Multiple observations support endothelial dysfunction as a central disease mechanism, including retinal arterial wall plaques and fluorescein leakage on angiography [3] and features of diffuse endothelial permeability on post‐gadolinium T2‐FLAIR MRI [4]. However, no established circulating biomarkers are currently available for SS. Anti‐endothelial cell antibodies show inconsistent pathogenic or diagnostic relevance, and while prior immunoblot studies identified SS‐associated protein bands, their precise molecular identification remains uncharacterized [5, 6].

We sought to determine if endothelial cell surface‐ or membrane‐associated proteins could serve as circulating biomarkers to differentiate SS cases from healthy controls. To this end, we performed mass spectrometry‐based proteomic analysis of serum, focusing comparisons on a pre‐defined panel of endothelial‐associated proteins with established expression in the brain, retina, and labyrinth microvasculature (Table S1).

## 2 Methods

### 2.1 Standard Protocol Approvals, Registrations, and Patient Consents

This study was conducted using samples and clinical data obtained prospectively from participants enrolled in two ongoing Institutional Review Board–approved natural history research protocols at the National Institutes of Health (ClinicalTrials.gov identifiers: NCT00001248 and NCT02435810). Written informed consent was obtained from all participants prior to inclusion in the study.

Participants that met diagnostic criteria for SS were included [7]. A sequential timeline of organ‐specific symptoms, investigations, and treatments was made to illustrate the fluctuating clinical course, using the modified Rankin Scale retrospectively to illustrate periods of worsening and recovery.

### 2.2 Proteomic Profiling

Proteomic analysis was performed on serum from SS cases and healthy controls. Following enrichment for extracellular vesicles, samples underwent data‐independent acquisition mass spectrometry on a high‐resolution Orbitrap platform [8]. Protein identification and quantification were performed using Spectronaut against the UniProt human reference proteome (UP000005640), with downstream processing using Protpipe [9]. In line with the study hypothesis, the unfiltered proteome was curated to select proteins with known endothelial cell surface—and membrane‐association expressed in brain, retinal, and labyrinthine microvasculature (Table S1).

### 2.3 Statistical Analysis

Proteomic data were analyzed using a two‐stage framework designed to address missingness inherent to mass spectrometry datasets. Detection frequencies were compared between groups using Fisher's exact test. Quantitative differences in protein abundance were assessed using probabilistic dropout analysis, which models intensity‐dependent missing values within a linear modeling framework. Results from both approaches were integrated into a prioritization score by summing the –log_10_‐transformed *p*‐values from each analysis. Statistical analyses were performed in R, and data were visualized using volcano and box‐and‐jitter plots.

## 3 Results

**FIGURE 1:**
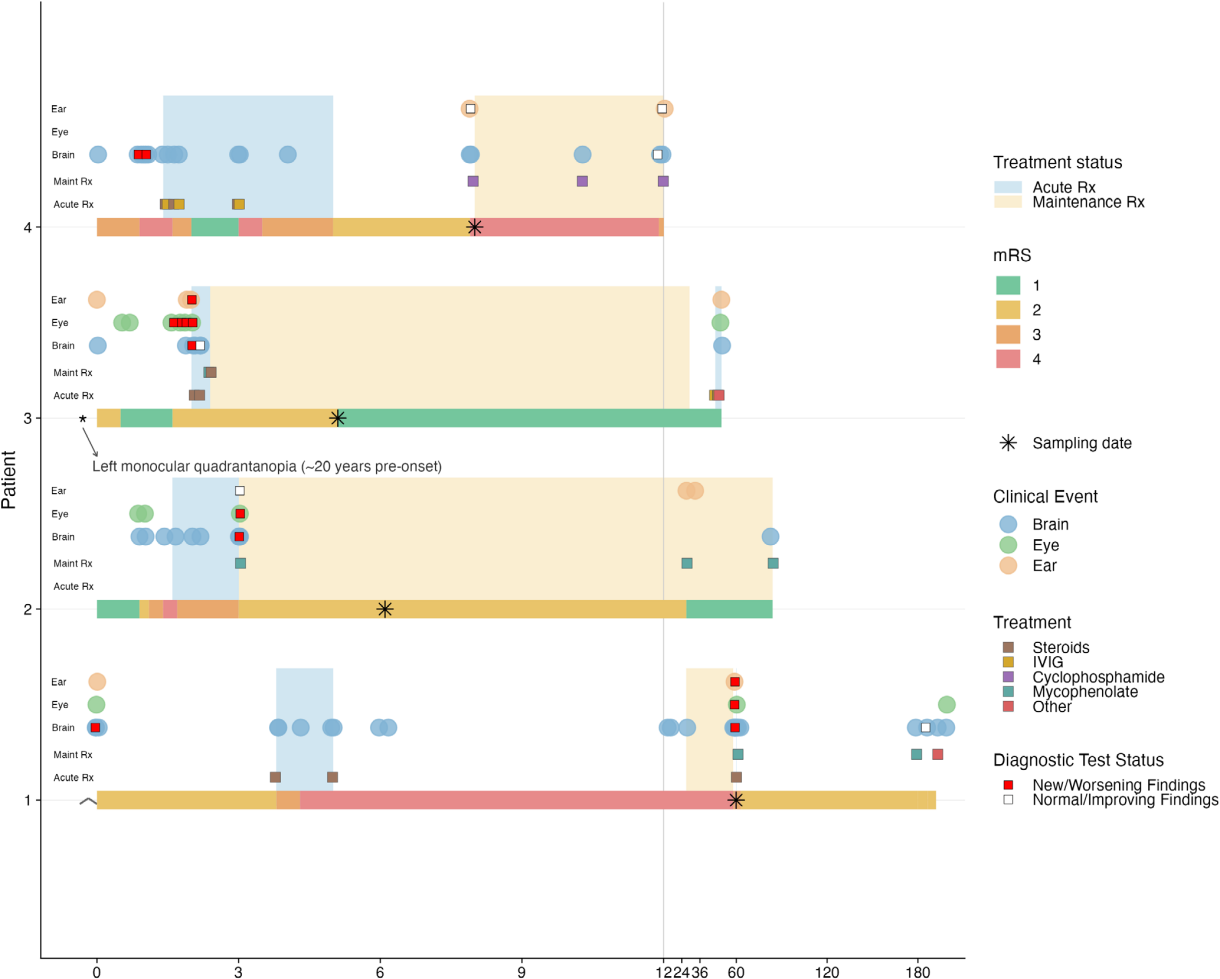
Swimmer Plot of longitudinal clinical course, organ involvement, diagnostic findings, and treatments in four patients with Susac syndrome. This visualization depicts the clinical timeline from onset of symptoms: Modified Rankin Scale (mRS) scores were applied retrospectively to historical clinical narratives to map functional disability synchronously with clinical target organ‐related events, pharmacologic treatments, and key diagnostic results (MRI Brain, Ophthalmology with Fluorescein Angio, and Audiometry for brain, eye, and ear, respectively). Shaded bands depict periods of being on Acute (corticosteroids/Intravenous immunoglobulin) or Maintenance immunosuppressive treatments. A remote neurological event for Patient 3 was monocular left upper nasal quadrant visual loss preceding the symptoms by 20 years.

Three cases had definite SS, and one had probable SS, with characteristic findings including central callosal lesions and post‐gadolinium T2‐FLAIR leptomeningeal enhancement on MRI Brain (Figure S1), capillary nonperfusion and arterial wall hyperfluorescence on fluorescein angiography, and sensorineural hearing loss (Table S2). The chronological timeline is summarized in the Swimmer plot (Figure 1).

Serum samples were obtained at variable time points relative to disease course, with a median interval of 7.1 months from symptom onset and 4.1 months from the most recent flare. All patients had received prior acute immunotherapy, and treatment status at the time of sampling varied, including maintenance immunosuppression in a subset of cases (Table S3).

Following endothelial filtering of the global proteomic dataset, 59 endothelial surface‐ or membrane‐associated proteins were quantified in serum (Table S4). Qualitative (Fisher's) analysis did not identify differences in detection frequencies between groups.

**FIGURE 2:**
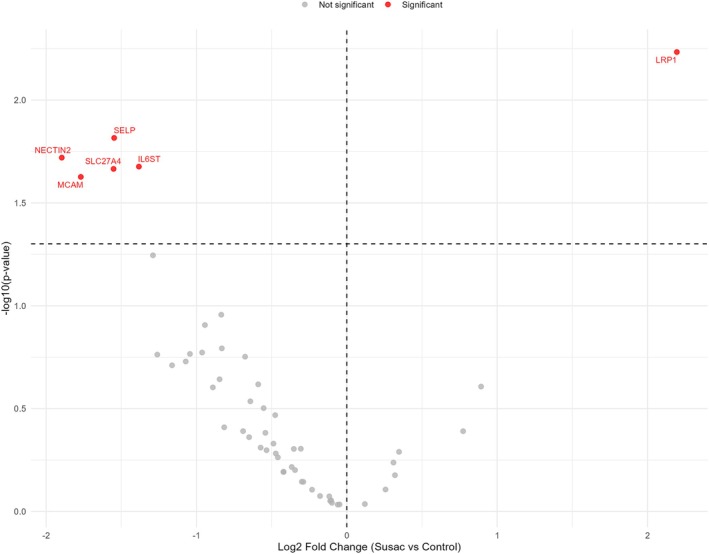
Differential protein expression between Susac syndrome and control samples. Volcano plot showing log2 fold change (Susac vs. control) on the x‐axis and − log10 (*p*‐value) on the y‐axis. IL6ST—Interleukin 6 signal transducer; LRP1—Low density lipoprotein receptor–related protein 1; MCAM—Melanoma cell adhesion molecule; NECTIN2—Nectin cell adhesion molecule 2; SELP—Selectin P; SLC27A4—Solute carrier family 27 member 4.

**FIGURE 3:**
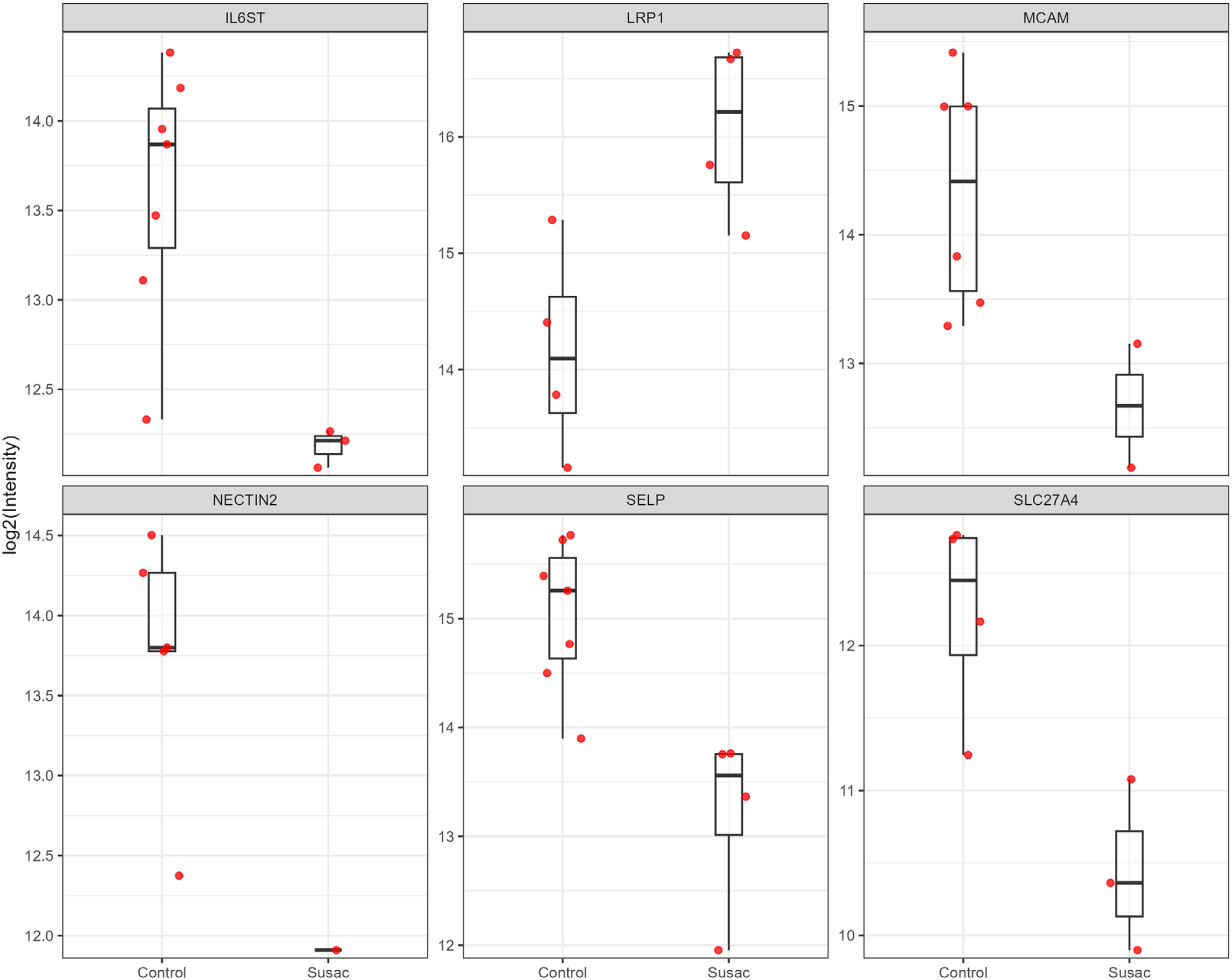
Differential abundance of selected proteins in Susac syndrome versus controls. Box‐and‐whisker plots showing log2‐transformed serum protein intensities for proteins found significant on quantitative analysis. LRP1 was increased in Susac samples, whereas IL6ST, MCAM, NECTIN2, SELP, and SLC27A4 showed reduced abundance compared with controls. Binary comparisons using the Wilcoxon rank‐sum test are included.

Quantitative analysis accounting for intensity‐dependent missingness identified six endothelial‐associated proteins with nominal differences in abundance in SS compared with controls (Figure 2). Low‐density lipoprotein receptor–related protein 1 (LRP1) was increased (log_2_ fold change = +2.19, *p* = 0.0059), whereas nectin cell adhesion molecule 2 (NECTIN2; −1.89, *p* = 0.0195), melanoma cell adhesion molecule (MCAM; −1.76, *p* = 0.0237), solute carrier family 27 member 4 (SLC27A4; −1.55, *p* = 0.0218), P‐selectin (SELP; −1.55, *p* = 0.0153), and interleukin‐6 signal transducer (IL6ST; −1.38, *p* = 0.0213) were decreased. No proteins met significance after correction for multiple testing. Integration of qualitative and quantitative analyses using a combined ranking metric did not alter the identity of the top candidates. Individual protein abundance distributions in serum and cerebrospinal fluid are shown in Figure 3.

## 4 Discussion

In this exploratory proteomic study, we identified six serum endothelial cell‐associated proteins that differed between SS cases and healthy controls. None of the observed differences met statistical significance after adjustment for multiple testing, reflecting the limited sample size and exploratory nature of this study. However, the six identified protein candidates demonstrate coherence with the pathology of SS and are described below. These features support the biological plausibility of the observed signals and justify further evaluation in larger, independent cohorts at varying disease stages.

The functions of these six proteins relate to endothelial integrity, leukocyte–endothelial cell interactions, or inflammatory signaling, thus reflecting the endothelial injury and barrier dysfunction seen in SS. Notably, these signals were detected despite most cases being sampled outside the acute disease phase and following exposure to immunotherapy, suggesting that they may reflect persistent endothelial alterations rather than transient inflammatory responses.

The most prominent finding was increased circulating low‐density lipoprotein receptor–related protein 1 (LRP1), a multifunctional endothelial receptor that maintains vascular stability by regulating TGF‐β signaling, protease clearance, and tight‐junction integrity [10]. Experimental loss of endothelial LRP1 disrupts blood–brain barrier structure and increases permeability [11], suggesting that the observed elevation here may reflect a compensatory response to endothelial injury or vascular repair rather than primary pathogenic activation.

In contrast, several adhesion and signaling molecules—including melanoma cell adhesion molecule (MCAM), nectin cell adhesion molecule 2 (NECTIN2), and P‐selectin (SELP) —were reduced. MCAM (CD146) regulates endothelial permeability and leukocyte transmigration and is elevated in active inflammatory vasculopathies [12, 13]. Reduced circulating MCAM may reflect post‐treatment suppression of endothelial activation or the sequestration of activated endothelial cells within the affected microvasculature. NECTIN2, a junctional adhesion molecule involved in barrier integrity and immune checkpoint interactions, may similarly reflect altered endothelial–immune cell crosstalk in convalescent disease [14].

Decreased interleukin‐6 signal transducer (IL6ST; gp130), the signal‐transducing subunit of the interleukin‐6 receptor complex that activates JAK–STAT signaling, suggests modulation of cytokine‐driven endothelial activation pathways, possibly after immunosuppressive therapy. The role of SLC27A4 in SS is less clear but may relate to endothelial metabolic stress responses during inflammation [15].

An important unanswered question is whether these endothelial cell protein changes are specific to Susac syndrome or represent shared responses to endothelial injury across inflammatory vasculopathies. The present study was not designed to address disease specificity, as comparator cohorts with primary CNS vasculitis or systemic vasculitis were not available. Furthermore, protein expression is likely to vary according to disease stage, treatment exposure, and the degree of ongoing endothelial injury. Accordingly, prospective studies incorporating standardized longitudinal sampling together with vasculitic and other inflammatory CNS comparator cohorts will be essential to distinguish disease‐specific biomarkers from more general markers of endothelial activation, injury, or repair.

Several additional limitations should be acknowledged. The cohort was small, and most cases were sampled during clinically stable or treatment phases, which may have attenuated disease‐associated signals and limited the utility of these proteins as diagnostic biomarkers of untreated disease. CSF samples were insufficient for formal analysis, limiting compartment‐specific conclusions. Accordingly, these findings should be interpreted as hypothesis‐generating and require further validation.

Despite these limitations, the results support the concept that circulating endothelial cell‐associated proteins may reflect disease‐related vascular biology in SS. Longitudinal sampling across the acute illness and treatment phases, and comparison with other inflammatory central nervous system disorders, will be important for determining disease specificity and mechanistic relevance.

## Author Contributions

**Rohit Benjamin:** conceptualization, methodology, visualization, software, writing – original draft. **Yue Andy Qi:** methodology, data curation, validation, writing – original draft, review and editing. **Ying Hao:** methodology, data curation. **Catherine DeMarino:** writing – original, review and editing, data curation, project administration. **Samuel Darko:** software, formal analysis, visualization, methodology. **Irene Cortese:** conceptualization, investigation, data curation, methodology. **Daniel S. Reich:** data curation, resources, writing – review amd editing, investigation. **María I. Gaitán:** data curation, writing – review and editing, investigation. **Steven Jacobson:** project administration, resources. **Bridgett J. Billioux:** data curation, investigation. **Bryan Smith:** data curation, investigation. **Avindra Nath:** conceptualization, investigation, data curation, funding acquisition, writing – review and editing, methodology, supervision, resources.

## Funding

This research was supported by the Division of Intramural Research of the NIH, NINDS (NS3130). The content is solely the responsibility of the author(s) and does not necessarily represent the official views of the National Institutes of Health or Health and Human Services.

## Conflicts of Interest

The authors declare no conflicts of interest.

## Supporting information


**Table S1:** Curated endothelial‐associated proteins and evidence for expression in brain, retinal, and labyrinthine vascular beds.
**Table S2:** Clinical characteristics of patients with Susac syndrome.
**Table S3:** Timing of sample collection in relation to disease course and treatment.
**Table S4:** Qualitative and quantitative analyses of endothelial‐associated proteins detected by mass spectrometry.
**Figure S1:** acn370508‐sup‐0001‐Supinfo.docx. *MRI Brain with diffuse endothelial gadolinium leakage in a patient with Susac Syndrome*. Axial and sagittal T2‐FLAIR images (A, B), post‐gadolinium T2‐FLAIR images (C, D), and post‐gadolinium T1‐weighted images (E, F) are shown. A central callosal lesion is indicated by the white arrow. Additional parenchymal lesions demonstrate a punched‐out appearance on post‐gadolinium T1‐weighted imaging. Post‐gadolinium T2‐FLAIR images demonstrate nodular leptomeningeal and perivascular enhancement involving cerebrospinal fluid–filled Virchow–Robin spaces, indicated by the circle. FLAIR = fluid‐attenuated inversion recovery; MRI = magnetic resonance imaging.

## Data Availability

Anonymized data not published within this article will be made available by request from any qualified investigator for purposes of replicating procedures and results.
