# Supplementary material for: Endothelial Cell Proteins as Biomarkers in Susac Syndrome
Source: Ann Clin Transl Neurol. 2026 Aug 13:10.1002/acn3.70508. Online ahead of print. doi: 10.1002/acn3.70508 (PMC13471125; doi:10.1002/acn3.70508)
Supplement: Supplementary file 1 [file ACN3-9999-0-s001.docx]

### **Table S1: Curated endothelial-associated proteins and evidence for expression in brain, retinal, and labyrinthine vascular beds.**

Candidate proteins are classified by gene symbol, protein name, functional category, subtype, and membrane topology. Evidence supporting expression in the blood–brain barrier, retinal vasculature, and labyrinthine microvasculature is qualitatively categorized as strong, moderate, limited, or unknown. The final column summarizes the principal endothelial vascular-bed function or context. BBB = blood–brain barrier; EC = endothelial cell; GPCR = G-protein–coupled receptor; GPI = glycosylphosphatidylinositol; IgSF = immunoglobulin superfamily; LDLR = low-density lipoprotein receptor; RTK = receptor tyrosine kinase; SLC = solute carrier.

| **Gene Symbol** | **Protein Name** | **Category** | **Subtype** | **Surface Topology** | **Evidence for BBB expression** | **Evidence for retinal expression** | **Evidence for labyrinth expression** | **Vascular Bed** |
| --- | --- | --- | --- | --- | --- | --- | --- | --- |
| **CAV1** | Caveolin-1 | Membrane scaffold | Caveolae | Membrane-associated | Strong | Strong | Strong | Vesicular transport & signaling |
| **CAV2** | Caveolin-2 | Membrane scaffold | Caveolae | Membrane-associated | Strong | Strong | Strong | Vesicular transport & signaling |
| **CDH5** | VE-cadherin (Cadherin-5) | Adhesion/Junction | IgSF/Cadherin/Claudin/etc. | Single-pass / multi-pass | Strong | Strong | Strong | Pan-endothelial; junctional/adhesion |
| **CLDN5** | Claudin-5 | Adhesion/Junction | IgSF/Cadherin/Claudin/etc. | Single-pass / multi-pass | Strong | Strong | Strong | Pan-endothelial; junctional/adhesion |
| **FLT1** | VEGFR1 | Growth factor / Guidance | RTK/Co-receptor | Single-pass receptor | Strong | Strong | Strong | Endothelial development/angiogenesis/vascular tone |
| **KDR** | VEGFR2 | Growth factor / Guidance | RTK/Co-receptor | Single-pass receptor | Strong | Strong | Strong | Endothelial development/angiogenesis/vascular tone |
| **OCLN** | Occludin | Adhesion/Junction | IgSF/Cadherin/Claudin/etc. | Single-pass / multi-pass | Strong | Strong | Strong | Pan-endothelial; junctional/adhesion |
| **PECAM1** | Platelet/endothelial cell adhesion molecule-1 (CD31) | Adhesion/Junction | IgSF/Cadherin/Claudin/etc. | Single-pass / multi-pass | Strong | Strong | Strong | Pan-endothelial; junctional/adhesion |
| **SLC2A1** | GLUT1 | Transporters/Channels | SLC/ABC/Aquaporin | Multi-pass membrane | Strong | Strong | Strong | BBB and systemic EC transport |
| **TEK** | TIE2 | Growth factor / Guidance | RTK/Co-receptor | Single-pass receptor | Strong | Strong | Strong | Endothelial development/angiogenesis/vascular tone |
| **TIE1** | TIE1 | Growth factor / Guidance | RTK/Co-receptor | Single-pass receptor | Strong | Strong | Strong | Endothelial development/angiogenesis/vascular tone |
| **ABCB1** | P-glycoprotein (MDR1) | Transporters/Channels | SLC/ABC/Aquaporin | Multi-pass membrane | Strong | Strong | Unknown | BBB and systemic EC transport |
| **ABCG2** | BCRP | Transporters/Channels | SLC/ABC/Aquaporin | Multi-pass membrane | Strong | Strong | Unknown | BBB and systemic EC transport |
| **DLL4** | Delta-like ligand 4 | Developmental signaling | TGF-β/BMP/Notch | Single-pass receptor/ligand | Strong | Strong | Unknown | Arterial identity, tip/stalk decisions |
| **ESAM** | Endothelial cell-selective adhesion molecule | Adhesion/Junction | IgSF/Cadherin/Claudin/etc. | Single-pass / multi-pass | Strong | Strong | Unknown | Pan-endothelial; junctional/adhesion |
| **JAG1** | Jagged-1 | Developmental signaling | TGF-β/BMP/Notch | Single-pass receptor/ligand | Strong | Strong | Unknown | Arterial identity, tip/stalk decisions |
| **MFSD2A** | MFSD2A | Transporters/Channels | SLC/ABC/Aquaporin | Multi-pass membrane | Strong | Strong | Unknown | BBB and systemic EC transport |
| **NOTCH1** | Notch 1 | Developmental signaling | TGF-β/BMP/Notch | Single-pass receptor/ligand | Strong | Strong | Unknown | Arterial identity, tip/stalk decisions |
| **NOTCH4** | Notch 4 | Developmental signaling | TGF-β/BMP/Notch | Single-pass receptor/ligand | Strong | Strong | Unknown | Arterial identity, tip/stalk decisions |
| **NRP1** | Neuropilin-1 | Growth factor / Guidance | RTK/Co-receptor | Single-pass receptor | Strong | Strong | Unknown | Endothelial development/angiogenesis/vascular tone |
| **ROBO4** | Roundabout homolog 4 | Adhesion/Junction | IgSF/Cadherin/Claudin/etc. | Single-pass / multi-pass | Strong | Strong | Unknown | Pan-endothelial; junctional/adhesion |
| **SLC3A2** | CD98 heavy chain (4F2hc) | Transporters/Channels | SLC/ABC/Aquaporin | Multi-pass membrane | Strong | Strong | Unknown | BBB and systemic EC transport |
| **SLC7A5** | LAT1 | Transporters/Channels | SLC/ABC/Aquaporin | Multi-pass membrane | Strong | Strong | Unknown | BBB and systemic EC transport |
| **ICAM2** | Intercellular adhesion molecule-2 (CD102) | Leukocyte adhesion | IgSF/Selectin | Type I membrane | Strong | Moderate | Moderate | Inducible on activated endothelium (SELE/SELP/ICAM1/VCAM1) |
| **APLNR** | Apelin receptor (APJ) | GPCRs | Lipid/peptide/chemokine receptors | 7TM GPCR | Strong | Moderate | Unknown | Barrier control, angiogenesis, leukocyte trafficking |
| **JAM2** | Junctional adhesion molecule B | Adhesion/Junction | IgSF/Cadherin/Claudin/etc. | Single-pass / multi-pass | Strong | Unknown | Strong | Pan-endothelial; junctional/adhesion |
| **JAM3** | Junctional adhesion molecule C | Adhesion/Junction | IgSF/Cadherin/Claudin/etc. | Single-pass / multi-pass | Strong | Unknown | Strong | Pan-endothelial; junctional/adhesion |
| **S1PR1** | Sphingosine-1-phosphate receptor 1 | GPCRs | Lipid/peptide/chemokine receptors | 7TM GPCR | Strong | Unknown | Strong | Barrier control, angiogenesis, leukocyte trafficking |
| **ACE** | Angiotensin-converting enzyme (CD143) | Ectoenzyme/Ectonucleotidase | Peptidase/phosphatase | Type I membrane/GPI-anchored | Strong | Unknown | Unknown | Barrier & inflammation modulation |
| **ACKR1** | Duffy antigen receptor for chemokines (DARC) | GPCRs | Lipid/peptide/chemokine receptors | 7TM GPCR | Strong | Unknown | Unknown | Barrier control, angiogenesis, leukocyte trafficking |
| **ADGRA2** | GPR124 (ADGRA2) | GPCRs | Lipid/peptide/chemokine receptors | 7TM GPCR | Strong | Unknown | Unknown | Barrier control, angiogenesis, leukocyte trafficking |
| **ECSCR** | Endothelial cell surface expressed chemotaxis and apoptosis regulator | Endothelial-enriched | Receptors/phosphatases | Single-pass/receptor-type phosphatase | Strong | Unknown | Unknown | EC-enriched |
| **ENTPD1** | CD39 (ecto-nucleotidase) | Ectoenzyme/Ectonucleotidase | Peptidase/phosphatase | Type I membrane/GPI-anchored | Strong | Unknown | Unknown | Barrier & inflammation modulation |
| **GJA4** | Connexin 37 | Gap junctions | Connexins | Channel | Strong | Unknown | Unknown | Arterial/venous endothelium |
| **GJA5** | Connexin 40 | Gap junctions | Connexins | Channel | Strong | Unknown | Unknown | Arterial/venous endothelium |
| **NT5E** | CD73 (ecto-5'-nucleotidase) | Ectoenzyme/Ectonucleotidase | Peptidase/phosphatase | Type I membrane/GPI-anchored | Strong | Unknown | Unknown | Barrier & inflammation modulation |
| **PLXND1** | Plexin-D1 | Growth factor / Guidance | RTK/Co-receptor | Single-pass receptor | Strong | Unknown | Unknown | Endothelial development/angiogenesis/vascular tone |
| **SCARB1** | SR-BI | Transporters/Channels | SLC/ABC/Aquaporin | Multi-pass membrane | Strong | Unknown | Unknown | BBB and systemic EC transport |
| **SLC16A1** | MCT1 | Transporters/Channels | SLC/ABC/Aquaporin | Multi-pass membrane | Strong | Unknown | Unknown | BBB and systemic EC transport |
| **EPHA2** | Ephrin type-A receptor 2 | Growth factor / Guidance | RTK/Co-receptor | Single-pass receptor | Moderate | Strong | Unknown | Endothelial development/angiogenesis/vascular tone |
| **ACE2** | Angiotensin-converting enzyme 2 | Ectoenzyme/Ectonucleotidase | Peptidase/phosphatase | Type I membrane/GPI-anchored | Moderate | Moderate | Moderate | Barrier & inflammation modulation |
| **ACKR3** | Atypical chemokine receptor 3 (CXCR7) | GPCRs | Lipid/peptide/chemokine receptors | 7TM GPCR | Moderate | Moderate | Moderate | Barrier control, angiogenesis, leukocyte trafficking |
| **ANPEP** | Aminopeptidase N (CD13) | Ectoenzyme/Ectonucleotidase | Peptidase/phosphatase | Type I membrane/GPI-anchored | Moderate | Moderate | Moderate | Barrier & inflammation modulation |
| **AQP1** | Aquaporin-1 | Transporters/Channels | SLC/ABC/Aquaporin | Multi-pass membrane | Moderate | Moderate | Moderate | BBB and systemic EC transport |
| **CD34** | CD34 | Adhesion/Junction | IgSF/Cadherin/Claudin/etc. | Single-pass / multi-pass | Moderate | Moderate | Moderate | Pan-endothelial; junctional/adhesion |
| **CXCR4** | C-X-C chemokine receptor 4 | GPCRs | Lipid/peptide/chemokine receptors | 7TM GPCR | Moderate | Moderate | Moderate | Barrier control, angiogenesis, leukocyte trafficking |
| **DPP4** | Dipeptidyl peptidase-4 (CD26) | Ectoenzyme/Ectonucleotidase | Peptidase/phosphatase | Type I membrane/GPI-anchored | Moderate | Moderate | Moderate | Barrier & inflammation modulation |
| **EDNRB** | Endothelin receptor type B | GPCRs | Lipid/peptide/chemokine receptors | 7TM GPCR | Moderate | Moderate | Moderate | Barrier control, angiogenesis, leukocyte trafficking |
| **EMCN** | Endomucin | Adhesion/Junction | IgSF/Cadherin/Claudin/etc. | Single-pass / multi-pass | Moderate | Moderate | Moderate | Pan-endothelial; junctional/adhesion |
| **IL1R1** | Interleukin-1 receptor type 1 | Cytokine receptors | Class I/II/TNF/IFN | Single-pass receptor | Moderate | Moderate | Moderate | Endothelial inflammatory signaling |
| **IL6R** | Interleukin-6 receptor | Cytokine receptors | Class I/II/TNF/IFN | Single-pass receptor | Moderate | Moderate | Moderate | Endothelial inflammatory signaling |
| **IL6ST** | gp130 (IL6 signal transducer) | Cytokine receptors | Class I/II/TNF/IFN | Single-pass receptor | Moderate | Moderate | Moderate | Endothelial inflammatory signaling |
| **MCAM** | CD146 (MCAM) | Adhesion/Junction | IgSF/Cadherin/Claudin/etc. | Single-pass / multi-pass | Moderate | Moderate | Moderate | Pan-endothelial; junctional/adhesion |
| **S1PR3** | S1P receptor 3 | GPCRs | Lipid/peptide/chemokine receptors | 7TM GPCR | Moderate | Moderate | Moderate | Barrier control, angiogenesis, leukocyte trafficking |
| **TNFRSF1A** | TNF receptor 1 | Cytokine receptors | Class I/II/TNF/IFN | Single-pass receptor | Moderate | Moderate | Moderate | Endothelial inflammatory signaling |
| **TNFRSF1B** | TNF receptor 2 | Cytokine receptors | Class I/II/TNF/IFN | Single-pass receptor | Moderate | Moderate | Moderate | Endothelial inflammatory signaling |
| **TRPC1** | TRPC1 | Ion channels | K+/TRP channels | Channel | Moderate | Moderate | Moderate | Shear sensing, Ca2+ entry |
| **TRPC4** | TRPC4 | Ion channels | K+/TRP channels | Channel | Moderate | Moderate | Moderate | Shear sensing, Ca2+ entry |
| **TRPV4** | TRPV4 | Ion channels | K+/TRP channels | Channel | Moderate | Moderate | Moderate | Shear sensing, Ca2+ entry |
| **STAB1** | Stabilin-1 | Scavenger/Endocytic | Class A/B/LDLR family | Type I/III membrane | Moderate | Moderate | Limited | Sinusoidal ECs (LSEC) and systemic endothelium |
| **ALCAM** | Activated leukocyte cell adhesion molecule | Immunoglobulin superfamily | IgSF adhesion/immunoreceptor | Type I membrane | Moderate | Moderate | Unknown | Subset on ECs; others leukocyte-enriched |
| **ALPL** | Tissue-nonspecific alkaline phosphatase | Ectoenzyme/Ectonucleotidase | Peptidase/phosphatase | Type I membrane/GPI-anchored | Moderate | Moderate | Unknown | Barrier & inflammation modulation |
| **CLEC14A** | C-type lectin domain family 14 member A | Adhesion/Junction | IgSF/Cadherin/Claudin/etc. | Single-pass / multi-pass | Moderate | Moderate | Unknown | Pan-endothelial; junctional/adhesion |
| **EPHB4** | Ephrin type-B receptor 4 | Growth factor / Guidance | RTK/Co-receptor | Single-pass receptor | Moderate | Moderate | Unknown | Endothelial development/angiogenesis/vascular tone |
| **F2R** | Protease-activated receptor 1 (PAR1) | GPCRs | Lipid/peptide/chemokine receptors | 7TM GPCR | Moderate | Moderate | Unknown | Barrier control, angiogenesis, leukocyte trafficking |
| **F2RL1** | Protease-activated receptor 2 (PAR2) | GPCRs | Lipid/peptide/chemokine receptors | 7TM GPCR | Moderate | Moderate | Unknown | Barrier control, angiogenesis, leukocyte trafficking |
| **GPR4** | GPR4 (proton-sensing) | GPCRs | Lipid/peptide/chemokine receptors | 7TM GPCR | Moderate | Moderate | Unknown | Barrier control, angiogenesis, leukocyte trafficking |
| **LDLR** | Low-density lipoprotein receptor | Endocytic/Lipoprotein receptors | LDLR family | Type I membrane | Moderate | Moderate | Unknown | Lipoprotein uptake/signaling |
| **LRP1** | LDL receptor-related protein 1 | Scavenger/Endocytic | Class A/B/LDLR family | Type I/III membrane | Moderate | Moderate | Unknown | Sinusoidal ECs (LSEC) and systemic endothelium |
| **NECTIN2** | Nectin-2 | Immunoglobulin superfamily | IgSF adhesion/immunoreceptor | Type I membrane | Moderate | Moderate | Unknown | Subset on ECs; others leukocyte-enriched |
| **PVR** | Poliovirus receptor (CD155) | Immunoglobulin superfamily | IgSF adhesion/immunoreceptor | Type I membrane | Moderate | Moderate | Unknown | Subset on ECs; others leukocyte-enriched |
| **SLC1A5** | ASCT2 | Transporters/Channels | SLC/ABC/Aquaporin | Multi-pass membrane | Moderate | Moderate | Unknown | BBB and systemic EC transport |
| **SLC43A2** | LAT4 | Transporters/Channels | SLC/ABC/Aquaporin | Multi-pass membrane | Moderate | Moderate | Unknown | BBB and systemic EC transport |
| **VLDLR** | Very low-density lipoprotein receptor | Endocytic/Lipoprotein receptors | LDLR family | Type I membrane | Moderate | Moderate | Unknown | Lipoprotein uptake/signaling |
| **MSR1** | Macrophage scavenger receptor 1 | Scavenger/Endocytic | Class A/B/LDLR family | Type I/III membrane | Moderate | Limited | Limited | Sinusoidal ECs (LSEC) and systemic endothelium |
| **STAB2** | Stabilin-2 | Scavenger/Endocytic | Class A/B/LDLR family | Type I/III membrane | Moderate | Limited | Limited | Sinusoidal ECs (LSEC) and systemic endothelium |
| **ADAM10** | ADAM10 | Ectoenzyme/Ectonucleotidase | Peptidase/phosphatase | Type I membrane/GPI-anchored | Moderate | Unknown | Unknown | Barrier & inflammation modulation |
| **ADAM17** | ADAM17 (TACE) | Ectoenzyme/Ectonucleotidase | Peptidase/phosphatase | Type I membrane/GPI-anchored | Moderate | Unknown | Unknown | Barrier & inflammation modulation |
| **ADGRL4** | ELTD1 (Adhesion GPCR L4) | GPCRs | Lipid/peptide/chemokine receptors | 7TM GPCR | Moderate | Unknown | Unknown | Barrier control, angiogenesis, leukocyte trafficking |
| **CD46** | Membrane cofactor protein (MCP) | Complement regulation | Complement control/receptor | GPI-anchored/Type I | Moderate | Unknown | Unknown | Host protection on endothelium |
| **CD55** | Decay-accelerating factor (DAF) | Complement regulation | Complement control/receptor | GPI-anchored/Type I | Moderate | Unknown | Unknown | Host protection on endothelium |
| **CD59** | Protectin | Complement regulation | Complement control/receptor | GPI-anchored/Type I | Moderate | Unknown | Unknown | Host protection on endothelium |
| **CD93** | C1q receptor (CD93) | Adhesion/Junction | IgSF/Cadherin/Claudin/etc. | Single-pass / multi-pass | Moderate | Unknown | Unknown | Pan-endothelial; junctional/adhesion |
| **CR1** | Complement receptor 1 (CD35) | Complement regulation | Complement control/receptor | GPI-anchored/Type I | Moderate | Unknown | Unknown | Host protection on endothelium |
| **EDNRA** | Endothelin receptor type A | GPCRs | Lipid/peptide/chemokine receptors | 7TM GPCR | Moderate | Unknown | Unknown | Barrier control, angiogenesis, leukocyte trafficking |
| **ENPEP** | Glutamyl aminopeptidase (CD249) | Ectoenzyme/Ectonucleotidase | Peptidase/phosphatase | Type I membrane/GPI-anchored | Moderate | Unknown | Unknown | Barrier & inflammation modulation |
| **F3** | Tissue factor (CD142) | Hemostasis | Coagulation/anti-coagulation | Type I or GPI-anchored | Moderate | Unknown | Unknown | Surface hemostasis regulation |
| **IFNAR1** | Interferon alpha/beta receptor 1 | Cytokine receptors | Class I/II/TNF/IFN | Single-pass receptor | Moderate | Unknown | Unknown | Endothelial inflammatory signaling |
| **IFNAR2** | Interferon alpha/beta receptor 2 | Cytokine receptors | Class I/II/TNF/IFN | Single-pass receptor | Moderate | Unknown | Unknown | Endothelial inflammatory signaling |
| **IFNGR1** | Interferon gamma receptor 1 | Cytokine receptors | Class I/II/TNF/IFN | Single-pass receptor | Moderate | Unknown | Unknown | Endothelial inflammatory signaling |
| **IFNGR2** | Interferon gamma receptor 2 | Cytokine receptors | Class I/II/TNF/IFN | Single-pass receptor | Moderate | Unknown | Unknown | Endothelial inflammatory signaling |
| **LRP5** | LRP5 | Endocytic/Lipoprotein receptors | LDLR family | Type I membrane | Moderate | Unknown | Unknown | Lipoprotein uptake/signaling |
| **LRP6** | LRP6 | Endocytic/Lipoprotein receptors | LDLR family | Type I membrane | Moderate | Unknown | Unknown | Lipoprotein uptake/signaling |
| **LRP8** | ApoER2 | Scavenger/Endocytic | Class A/B/LDLR family | Type I/III membrane | Moderate | Unknown | Unknown | Sinusoidal ECs (LSEC) and systemic endothelium |
| **MADCAM1** | Mucosal vascular addressin (MAdCAM-1) | Adhesion/Junction | IgSF/Cadherin/Claudin/etc. | Single-pass / multi-pass | Moderate | Unknown | Unknown | Pan-endothelial; junctional/adhesion |
| **PLAU** | Urokinase-type plasminogen activator (ligand) | Hemostasis | Coagulation/anti-coagulation | Type I or GPI-anchored | Moderate | Unknown | Unknown | Surface hemostasis regulation |
| **PLAUR** | uPAR (CD87) | Hemostasis | Coagulation/anti-coagulation | Type I or GPI-anchored | Moderate | Unknown | Unknown | Surface hemostasis regulation |
| **PROCR** | Endothelial protein C receptor (EPCR) | Hemostasis | Coagulation/anti-coagulation | Type I or GPI-anchored | Moderate | Unknown | Unknown | Surface hemostasis regulation |
| **PTPRB** | VE-PTP | Endothelial-enriched | Receptors/phosphatases | Single-pass/receptor-type phosphatase | Moderate | Unknown | Unknown | EC-enriched |
| **PTPRJ** | CD148 | Endothelial-enriched | Receptors/phosphatases | Single-pass/receptor-type phosphatase | Moderate | Unknown | Unknown | EC-enriched |
| **SLC38A5** | SNAT5 | Transporters/Channels | SLC/ABC/Aquaporin | Multi-pass membrane | Moderate | Unknown | Unknown | BBB and systemic EC transport |
| **SLC47A1** | MATE1 | Transporters/Channels | SLC/ABC/Aquaporin | Multi-pass membrane | Moderate | Unknown | Unknown | BBB and systemic EC transport |
| **SLC6A6** | TauT | Transporters/Channels | SLC/ABC/Aquaporin | Multi-pass membrane | Moderate | Unknown | Unknown | BBB and systemic EC transport |
| **TFPI** | Tissue factor pathway inhibitor | Hemostasis | Coagulation/anti-coagulation | Type I or GPI-anchored | Moderate | Unknown | Unknown | Surface hemostasis regulation |
| **THBD** | Thrombomodulin (CD141) | Hemostasis | Coagulation/anti-coagulation | Type I or GPI-anchored | Moderate | Unknown | Unknown | Surface hemostasis regulation |
| **UNC5B** | Netrin receptor UNC5B | Growth factor / Guidance | RTK/Co-receptor | Single-pass receptor | Moderate | Unknown | Unknown | Endothelial development/angiogenesis/vascular tone |
| **PLVAP** | Plasmalemma vesicle-associated protein (PV1) | Fenestration/diaphragm | Diaphragm component | Type II membrane | Limited | Moderate | Limited | Fenestrated capillaries; absent at BBB |
| **CD151** | CD151 | Tetraspanins | Tetraspanin | 4-pass membrane | Limited | Limited | Limited | Microdomain organization on ECs |
| **CD81** | CD81 | Tetraspanins | Tetraspanin | 4-pass membrane | Limited | Limited | Limited | Microdomain organization on ECs |
| **CD9** | CD9 | Tetraspanins | Tetraspanin | 4-pass membrane | Limited | Limited | Limited | Microdomain organization on ECs |
| **GPC1** | Glypican-1 | Glycocalyx | Heparan sulfate proteoglycans | Type I or GPI-anchored | Limited | Limited | Limited | Endothelial glycocalyx |
| **ITGA1** | Integrin alpha-1 | Integrins | Alpha subunit | Type I membrane | Limited | Limited | Limited | Subset expressed on ECs (e.g., α5, αV, α9) |
| **ITGA10** | Integrin alpha-10 | Integrins | Alpha subunit | Type I membrane | Limited | Limited | Limited | Subset expressed on ECs (e.g., α5, αV, α9) |
| **ITGA11** | Integrin alpha-11 | Integrins | Alpha subunit | Type I membrane | Limited | Limited | Limited | Subset expressed on ECs (e.g., α5, αV, α9) |
| **ITGA2** | Integrin alpha-2 | Integrins | Alpha subunit | Type I membrane | Limited | Limited | Limited | Subset expressed on ECs (e.g., α5, αV, α9) |
| **ITGA2B** | Integrin alpha-IIb | Integrins | Alpha subunit | Type I membrane | Limited | Limited | Limited | Subset expressed on ECs (e.g., α5, αV, α9) |
| **ITGA3** | Integrin alpha-3 | Integrins | Alpha subunit | Type I membrane | Limited | Limited | Limited | Subset expressed on ECs (e.g., α5, αV, α9) |
| **ITGA4** | Integrin alpha-4 | Integrins | Alpha subunit | Type I membrane | Limited | Limited | Limited | Subset expressed on ECs (e.g., α5, αV, α9) |
| **ITGA5** | Integrin alpha-5 | Integrins | Alpha subunit | Type I membrane | Limited | Limited | Limited | Subset expressed on ECs (e.g., α5, αV, α9) |
| **ITGA6** | Integrin alpha-6 | Integrins | Alpha subunit | Type I membrane | Limited | Limited | Limited | Subset expressed on ECs (e.g., α5, αV, α9) |
| **ITGA7** | Integrin alpha-7 | Integrins | Alpha subunit | Type I membrane | Limited | Limited | Limited | Subset expressed on ECs (e.g., α5, αV, α9) |
| **ITGA8** | Integrin alpha-8 | Integrins | Alpha subunit | Type I membrane | Limited | Limited | Limited | Subset expressed on ECs (e.g., α5, αV, α9) |
| **ITGA9** | Integrin alpha-9 | Integrins | Alpha subunit | Type I membrane | Limited | Limited | Limited | Subset expressed on ECs (e.g., α5, αV, α9) |
| **ITGAD** | Integrin alpha-D | Integrins | Alpha subunit | Type I membrane | Limited | Limited | Limited | Subset expressed on ECs (e.g., α5, αV, α9) |
| **ITGAE** | Integrin alpha-E | Integrins | Alpha subunit | Type I membrane | Limited | Limited | Limited | Subset expressed on ECs (e.g., α5, αV, α9) |
| **ITGAL** | Integrin alpha-L | Integrins | Alpha subunit | Type I membrane | Limited | Limited | Limited | Subset expressed on ECs (e.g., α5, αV, α9) |
| **ITGAM** | Integrin alpha-M | Integrins | Alpha subunit | Type I membrane | Limited | Limited | Limited | Subset expressed on ECs (e.g., α5, αV, α9) |
| **ITGAV** | Integrin alpha-V | Integrins | Alpha subunit | Type I membrane | Limited | Limited | Limited | Subset expressed on ECs (e.g., α5, αV, α9) |
| **ITGAX** | Integrin alpha-X | Integrins | Alpha subunit | Type I membrane | Limited | Limited | Limited | Subset expressed on ECs (e.g., α5, αV, α9) |
| **ITGB1** | Integrin beta-1 | Integrins | Beta subunit | Type I membrane | Limited | Limited | Limited | Common EC partners β1, β3, β5 |
| **ITGB2** | Integrin beta-2 | Integrins | Beta subunit | Type I membrane | Limited | Limited | Limited | Common EC partners β1, β3, β5 |
| **ITGB3** | Integrin beta-3 | Integrins | Beta subunit | Type I membrane | Limited | Limited | Limited | Common EC partners β1, β3, β5 |
| **ITGB4** | Integrin beta-4 | Integrins | Beta subunit | Type I membrane | Limited | Limited | Limited | Common EC partners β1, β3, β5 |
| **ITGB5** | Integrin beta-5 | Integrins | Beta subunit | Type I membrane | Limited | Limited | Limited | Common EC partners β1, β3, β5 |
| **ITGB6** | Integrin beta-6 | Integrins | Beta subunit | Type I membrane | Limited | Limited | Limited | Common EC partners β1, β3, β5 |
| **ITGB7** | Integrin beta-7 | Integrins | Beta subunit | Type I membrane | Limited | Limited | Limited | Common EC partners β1, β3, β5 |
| **ITGB8** | Integrin beta-8 | Integrins | Beta subunit | Type I membrane | Limited | Limited | Limited | Common EC partners β1, β3, β5 |
| **SDC1** | Syndecan-1 | Glycocalyx | Heparan sulfate proteoglycans | Type I or GPI-anchored | Limited | Limited | Limited | Endothelial glycocalyx |
| **SDC2** | Syndecan-2 | Glycocalyx | Heparan sulfate proteoglycans | Type I or GPI-anchored | Limited | Limited | Limited | Endothelial glycocalyx |
| **SDC3** | Syndecan-3 | Glycocalyx | Heparan sulfate proteoglycans | Type I or GPI-anchored | Limited | Limited | Limited | Endothelial glycocalyx |
| **SDC4** | Syndecan-4 | Glycocalyx | Heparan sulfate proteoglycans | Type I or GPI-anchored | Limited | Limited | Limited | Endothelial glycocalyx |
| **TSPAN12** | Tetraspanin-12 | Tetraspanins | Tetraspanin | 4-pass membrane | Limited | Limited | Limited | Microdomain organization on ECs |
| **TSPAN7** | Tetraspanin-7 | Tetraspanins | Tetraspanin | 4-pass membrane | Limited | Limited | Limited | Microdomain organization on ECs |
| **ICAM1** | Intercellular adhesion molecule-1 (CD54) | Leukocyte adhesion | IgSF/Selectin | Type I membrane | Unknown | Strong | Strong | Inducible on activated endothelium (SELE/SELP/ICAM1/VCAM1) |
| **VCAM1** | Vascular cell adhesion molecule-1 (CD106) | Leukocyte adhesion | IgSF/Selectin | Type I membrane | Unknown | Strong | Strong | Inducible on activated endothelium (SELE/SELP/ICAM1/VCAM1) |
| **SELE** | E-selectin (CD62E) | Leukocyte adhesion | IgSF/Selectin | Type I membrane | Unknown | Moderate | Moderate | Inducible on activated endothelium (SELE/SELP/ICAM1/VCAM1) |
| **SELP** | P-selectin (CD62P) | Leukocyte adhesion | IgSF/Selectin | Type I membrane | Unknown | Moderate | Moderate | Inducible on activated endothelium (SELE/SELP/ICAM1/VCAM1) |
| **ACVRL1** | ALK 1.00 | Developmental signaling | TGF-β/BMP/Notch | Single-pass receptor/ligand | Unknown | Unknown | Unknown | Arterial identity, tip/stalk decisions |
| **AXL** | AXL receptor tyrosine kinase | Growth factor / Guidance | RTK/Co-receptor | Single-pass receptor | Unknown | Unknown | Unknown | Endothelial development/angiogenesis/vascular tone |
| **BMPR1A** | BMP receptor type IA | Developmental signaling | TGF-β/BMP/Notch | Single-pass receptor/ligand | Unknown | Unknown | Unknown | Arterial identity, tip/stalk decisions |
| **BMPR1B** | BMP receptor type IB | Developmental signaling | TGF-β/BMP/Notch | Single-pass receptor/ligand | Unknown | Unknown | Unknown | Arterial identity, tip/stalk decisions |
| **BMPR2** | BMP receptor type II | Developmental signaling | TGF-β/BMP/Notch | Single-pass receptor/ligand | Unknown | Unknown | Unknown | Arterial identity, tip/stalk decisions |
| **CD36** | CD36 | Scavenger/Endocytic | Class A/B/LDLR family | Type I/III membrane | Unknown | Unknown | Unknown | Sinusoidal ECs (LSEC) and systemic endothelium |
| **CD99** | CD99 | Adhesion/Junction | IgSF/Cadherin/Claudin/etc. | Single-pass / multi-pass | Unknown | Unknown | Unknown | Pan-endothelial; junctional/adhesion |
| **CDH13** | T-cadherin (GPI-anchored) | Cell–cell junction | Cadherin/Nectin | Type I or GPI-anchored | Unknown | Unknown | Unknown | Endothelial junctional complexes |
| **CDH2** | N-cadherin (EC-Pericyte contacts) | Cell–cell junction | Cadherin/Nectin | Type I or GPI-anchored | Unknown | Unknown | Unknown | Endothelial junctional complexes |
| **DLL1** | Delta-like ligand 1 | Developmental signaling | TGF-β/BMP/Notch | Single-pass receptor/ligand | Unknown | Unknown | Unknown | Arterial identity, tip/stalk decisions |
| **ENG** | Endoglin | Developmental signaling | TGF-β/BMP/Notch | Single-pass receptor/ligand | Unknown | Unknown | Unknown | Arterial identity, tip/stalk decisions |
| **FGFR1** | Fibroblast growth factor receptor 1 | Growth factor / Guidance | RTK/Co-receptor | Single-pass receptor | Unknown | Unknown | Unknown | Endothelial development/angiogenesis/vascular tone |
| **FGFR2** | Fibroblast growth factor receptor 2 | Growth factor / Guidance | RTK/Co-receptor | Single-pass receptor | Unknown | Unknown | Unknown | Endothelial development/angiogenesis/vascular tone |
| **FLT4** | VEGFR3 | Growth factor / Guidance | RTK/Co-receptor | Single-pass receptor | Unknown | Unknown | Unknown | Endothelial development/angiogenesis/vascular tone |
| **GJC1** | Connexin 45 | Gap junctions | Connexins | Channel | Unknown | Unknown | Unknown | Arterial/venous endothelium |
| **GPC4** | Glypican-4 | Glycocalyx | Heparan sulfate proteoglycans | Type I or GPI-anchored | Unknown | Unknown | Unknown | Endothelial glycocalyx |
| **GPC6** | Glypican-6 | Glycocalyx | Heparan sulfate proteoglycans | Type I or GPI-anchored | Unknown | Unknown | Unknown | Endothelial glycocalyx |
| **GPR182** | GPR182 (ADGRF4) | GPCRs | Lipid/peptide/chemokine receptors | 7TM GPCR | Unknown | Unknown | Unknown | Barrier control, angiogenesis, leukocyte trafficking |
| **ICAM3** | Intercellular adhesion molecule-3 (CD50) | Leukocyte adhesion | IgSF/Selectin | Type I membrane | Unknown | Unknown | Unknown | Inducible on activated endothelium (SELE/SELP/ICAM1/VCAM1) |
| **INSR** | Insulin receptor | Growth factor / Guidance | RTK/Co-receptor | Single-pass receptor | Unknown | Unknown | Unknown | Endothelial development/angiogenesis/vascular tone |
| **JAG2** | Jagged-2 | Developmental signaling | TGF-β/BMP/Notch | Single-pass receptor/ligand | Unknown | Unknown | Unknown | Arterial identity, tip/stalk decisions |
| **KCNJ8** | Kir6.1 | Ion channels | K+/TRP channels | Channel | Unknown | Unknown | Unknown | Shear sensing, Ca2+ entry |
| **KCNK3** | TASK-1 (K2P3.1) | Ion channels | K+/TRP channels | Channel | Unknown | Unknown | Unknown | Shear sensing, Ca2+ entry |
| **LRP2** | Megalin | Endocytic/Lipoprotein receptors | LDLR family | Type I membrane | Unknown | Unknown | Unknown | Lipoprotein uptake/signaling |
| **LY75** | DEC-205 (CD205) | Scavenger/Endocytic | Class A/B/LDLR family | Type I/III membrane | Unknown | Unknown | Unknown | Sinusoidal ECs (LSEC) and systemic endothelium |
| **LYVE1** | Lymphatic vessel endothelial hyaluronan receptor-1 | Lymphatic endothelium | Mucin-type/Hyaluronan receptor | Type I membrane | Unknown | Unknown | Unknown | Lymphatic ECs |
| **NECTIN1** | Nectin-1 | Cell–cell junction | Cadherin/Nectin | Type I or GPI-anchored | Unknown | Unknown | Unknown | Endothelial junctional complexes |
| **NECTIN3** | Nectin-3 | Cell–cell junction | Cadherin/Nectin | Type I or GPI-anchored | Unknown | Unknown | Unknown | Endothelial junctional complexes |
| **NRP2** | Neuropilin-2 | Growth factor / Guidance | RTK/Co-receptor | Single-pass receptor | Unknown | Unknown | Unknown | Endothelial development/angiogenesis/vascular tone |
| **OLR1** | LOX-1 | Scavenger/Endocytic | Class A/B/LDLR family | Type I/III membrane | Unknown | Unknown | Unknown | Sinusoidal ECs (LSEC) and systemic endothelium |
| **P2RX4** | P2X4 | Purinergic receptors | GPCR/Ligand-gated ion channels | 7TM/Channel | Unknown | Unknown | Unknown | Shear/mechanotransduction; inflammation |
| **P2RX7** | P2X7 | Purinergic receptors | GPCR/Ligand-gated ion channels | 7TM/Channel | Unknown | Unknown | Unknown | Shear/mechanotransduction; inflammation |
| **P2RY1** | P2Y1 | Purinergic receptors | GPCR/Ligand-gated ion channels | 7TM/Channel | Unknown | Unknown | Unknown | Shear/mechanotransduction; inflammation |
| **P2RY2** | P2Y2 | Purinergic receptors | GPCR/Ligand-gated ion channels | 7TM/Channel | Unknown | Unknown | Unknown | Shear/mechanotransduction; inflammation |
| **PDGFRB** | Platelet-derived growth factor receptor beta | Growth factor / Guidance | RTK/Co-receptor | Single-pass receptor | Unknown | Unknown | Unknown | Endothelial development/angiogenesis/vascular tone |
| **PDPN** | Podoplanin (GP38) | Lymphatic endothelium | Mucin-type/Hyaluronan receptor | Type I membrane | Unknown | Unknown | Unknown | Lymphatic ECs |
| **PTRF** | CAVIN1 | Membrane scaffold | Caveolae | Membrane-associated | Unknown | Unknown | Unknown | Vesicular transport & signaling |
| **SDPR** | CAVIN2 | Membrane scaffold | Caveolae | Membrane-associated | Unknown | Unknown | Unknown | Vesicular transport & signaling |
| **SIGLEC1** | Sialoadhesin | Immunoglobulin superfamily | IgSF adhesion/immunoreceptor | Type I membrane | Unknown | Unknown | Unknown | Subset on ECs; others leukocyte-enriched |
| **SIGLEC7** | Siglec-7 | Immunoglobulin superfamily | IgSF adhesion/immunoreceptor | Type I membrane | Unknown | Unknown | Unknown | Subset on ECs; others leukocyte-enriched |
| **SLC12A2** | NKCC1 | Transporters | SLC family | Multi-pass | Unknown | Unknown | Unknown | Expression varies by vascular bed/species; not all EC-specific |
| **SLC12A7** | KCC4 | Transporters | SLC family | Multi-pass | Unknown | Unknown | Unknown | Expression varies by vascular bed/species; not all EC-specific |
| **SLC15A1** | PepT1 | Transporters | SLC family | Multi-pass | Unknown | Unknown | Unknown | Expression varies by vascular bed/species; not all EC-specific |
| **SLC15A2** | PepT2 | Transporters | SLC family | Multi-pass | Unknown | Unknown | Unknown | Expression varies by vascular bed/species; not all EC-specific |
| **SLC22A1** | OCT1 | Transporters | SLC family | Multi-pass | Unknown | Unknown | Unknown | Expression varies by vascular bed/species; not all EC-specific |
| **SLC22A2** | OCT2 | Transporters | SLC family | Multi-pass | Unknown | Unknown | Unknown | Expression varies by vascular bed/species; not all EC-specific |
| **SLC22A5** | OCTN2 | Transporters | SLC family | Multi-pass | Unknown | Unknown | Unknown | Expression varies by vascular bed/species; not all EC-specific |
| **SLC22A8** | OAT3 | Transporters | SLC family | Multi-pass | Unknown | Unknown | Unknown | Expression varies by vascular bed/species; not all EC-specific |
| **SLC27A1** | FATP1 | Transporters | SLC family | Multi-pass | Unknown | Unknown | Unknown | Expression varies by vascular bed/species; not all EC-specific |
| **SLC27A4** | FATP4 | Transporters | SLC family | Multi-pass | Unknown | Unknown | Unknown | Expression varies by vascular bed/species; not all EC-specific |
| **SLC2A3** | GLUT3 | Transporters/Channels | SLC/ABC/Aquaporin | Multi-pass membrane | Unknown | Unknown | Unknown | BBB and systemic EC transport |
| **SLC2A8** | GLUT8 | Transporters/Channels | SLC/ABC/Aquaporin | Multi-pass membrane | Unknown | Unknown | Unknown | BBB and systemic EC transport |
| **SLC30A10** | ZnT10 | Transporters | SLC family | Multi-pass | Unknown | Unknown | Unknown | Expression varies by vascular bed/species; not all EC-specific |
| **SLC39A8** | ZIP8 | Transporters | SLC family | Multi-pass | Unknown | Unknown | Unknown | Expression varies by vascular bed/species; not all EC-specific |
| **SLC7A1** | CAT-1 (cationic amino acid transporter 1) | Transporters/Channels | SLC/ABC/Aquaporin | Multi-pass membrane | Unknown | Unknown | Unknown | BBB and systemic EC transport |
| **SLC7A7** | y+LAT1 | Transporters | SLC family | Multi-pass | Unknown | Unknown | Unknown | Expression varies by vascular bed/species; not all EC-specific |
| **SLC7A8** | LAT2 | Transporters | SLC family | Multi-pass | Unknown | Unknown | Unknown | Expression varies by vascular bed/species; not all EC-specific |
| **TGFBR1** | TGF-β receptor type I | Developmental signaling | TGF-β/BMP/Notch | Single-pass receptor/ligand | Unknown | Unknown | Unknown | Arterial identity, tip/stalk decisions |
| **TGFBR2** | TGF-β receptor type II | Developmental signaling | TGF-β/BMP/Notch | Single-pass receptor/ligand | Unknown | Unknown | Unknown | Arterial identity, tip/stalk decisions |
| **TYRO3** | TYRO3 protein tyrosine kinase | Growth factor / Guidance | RTK/Co-receptor | Single-pass receptor | Unknown | Unknown | Unknown | Endothelial development/angiogenesis/vascular tone |

### **Table S2. Clinical characteristics of patients with Susac syndrome.**

### The table summarizes diagnostic classification and neurological, neuroimaging, ophthalmological, audiovestibular, and cerebrospinal fluid findings for the four included patients. BRAO = branch retinal artery occlusion; CSF = cerebrospinal fluid; FA = fluorescein angiography; FLAIR = fluid-attenuated inversion recovery; HL = hearing loss; IgG = immunoglobulin G; L = left; MRI = magnetic resonance imaging; R = right; WC = White Caucasian.

|  | **Patient 1** | **Patient 2** | **Patient 3** | **Patient 4** |
| --- | --- | --- | --- | --- |
| **Age category at onset [years]** | 35-40 | 20-25 | 40-45 | 20-25 |
| **Ethnicity** | WC | WC | WC | WC |
|  |  |  |  |  |
| **Diagnostic category** | Definite | Probable | Definite | Definite |
|  |  |  |  |  |
| **Neurological Involvement** | Yes | Yes | Yes | Yes |
| **Headache** | Yes | Yes | Yes | No |
| **Encephalopathy** | Yes | Yes | No | Yes |
| **Speech and language** | Yes | Yes | No | No |
| **Focal deficits** | Yes | No | No | Yes |
| **MRI T2-FLAIR white matter lesions** | Yes | Yes | Yes | Yes |
| **MRI Central callosal lesions** | Yes | Yes | Yes | Yes |
| **MRI T2-FLAIR post-gadolinium leptomeningeal enhancement** | Yes | Yes | Yes | Yes |
|  |  |  |  |  |
| **Ophthalmological involvement** | Yes | Yes | Yes | Yes |
| **Transient visual symptoms** | Yes | No | Yes | Yes |
| **Permanent residual scotoma** | No | Bilateral | New Right  Past Left | Bilateral |
| **BRAO** | No | None | R - acute  L- past | Bilateral |
| **Capillary nonperfusion on FA** | Bilateral | Bilateral | R | Bilateral |
| **Other findings** | Leakage Bilateral | Arterial Wall Hyper-Fluorescence | R Leakage | Bilateral fluorescein leak |
|  |  |  |  |  |
| **Audio-vestibular involvement** |  |  |  |  |
| **Peripheral vertigo** | Yes | No | No | No |
| **Tinnitus** | Yes | Yes | No | No |
| **Hearing loss** | Yes | No | Yes | Yes |
| **Audiology** | R-Normal  L- Mild HL | R:Normal  L:Normal | R: Normal  L: Mild HL 4000-6000Hz | R: Mod-Severe HL 250-3000 Hz  L: Mod-Severe HL 250-4000Hz |
|  |  |  |  |  |
| **CSF Cells (/cm.mm)** | 4 | 18 | 2 | 10 (L90%) |
| **CSF Protein (highest, mg/dl)** | 242.0 | 196.5 | 44.0 | 115.4 |
| **CSF Glucose (mg/dl)** | 45.0 | 60.0 | 51.0 | n/a |
| **CSF Oligoclonal bands** | Negative | Negative | Negative | Negative |
| **IgG Index** | 0.47 | n/a | 0.48 | n/a |

Abbreviations: L-Left R -Right; WC- White Caucasian

**Table S3. Timing of sample collection in relation to disease course and treatment.**

For each patient, the table shows the interval from symptom onset and the most recent clinical flare to serum collection, together with acute immunotherapy received before sampling and maintenance immunosuppressive therapy at the time of sampling. CP = cyclophosphamide; CS = corticosteroids; IVIG = intravenous immunoglobulin; MMF = mycophenolate mofetil.

| **Patient** | **Months from onset to sampling** | **Months from last flare to sampling** | **Acute treatment prior to sampling** | **Maintenance therapy at sampling** |
| --- | --- | --- | --- | --- |
| **1** | 59.9 | 0.3 | Yes (CS) | Yes (CP – One Pulse) |
| **2** | 6.1 | 6.1 | Yes (CS) | Yes (MMF) |
| **3** | 5.1 | 5.1 | Yes (CS) | Yes (MMF) |
| **4** | 8.0 | 3.0 | Yes (CS + IVIG) | No |

**Table S4. Qualitative and quantitative analyses of endothelial-associated proteins detected by mass spectrometry.**

The table presents protein-detection comparisons using Fisher’s exact test and quantitative abundance comparisons using probabilistic dropout analysis (proDA). Reported measures include the Fisher odds ratio and p value, false-discovery rate (FDR), average abundance, log₂ fold change for Susac syndrome versus healthy controls, proDA p value and FDR, and the combined prioritization score. The combined score was calculated as the sum of the −log₁₀-transformed Fisher and proDA p values. “Significant” denotes a nominal proDA p value <0.05; no protein remained significant after correction for multiple testing. Inf = infinity.

| **Genes** | **Fisher OddsRatio** | **Fisher p-value** | **Fisher FDR** | **Average abundance** | **log2FC** | **proDA p-value** | **proDA FDR** | **combined score** | **evidence class** |
| --- | --- | --- | --- | --- | --- | --- | --- | --- | --- |
| **LRP1** | Inf | 0.208 | 1 | 14.387 | 2.193 | **0.006** | 0.233 | 2.914 | Significant |
| **NECTIN2** | 0.230 | 0.545 | 1 | 12.857 | -1.888 | **0.020** | 0.233 | 1.972 | Significant |
| **SLC27A4** | 2.742 | 0.576 | 1 | 11.680 | -1.551 | **0.022** | 0.233 | 1.902 | Significant |
| **MCAM** | 0.369 | 0.547 | 1 | 13.317 | -1.764 | **0.024** | 0.233 | 1.886 | Significant |
| **SELP** | Inf | 1.000 | 1 | 14.259 | -1.547 | **0.015** | 0.233 | 1.817 | Significant |
| **IL6ST** | 0.463 | 1.000 | 1 | 13.111 | -1.381 | **0.021** | 0.233 | 1.673 | Significant |
| **ESAM** | 0.463 | 1.000 | 1 | 13.598 | -1.288 | 0.057 | 0.480 | 1.245 | Not significant |
| **CD99** | 0.139 | 0.222 | 1 | 12.159 | -0.532 | 0.423 | 0.832 | 1.027 | Not significant |
| **ALCAM** | 0.000 | 0.515 | 1 | 11.754 | -1.059 | 0.193 | 0.733 | 1.004 | Not significant |
| **CDH13** | Inf | 1.000 | 1 | 14.376 | -0.835 | 0.111 | 0.733 | 0.956 | Not significant |
| **ITGA5** | 1.000 | 1.000 | 1 | 13.155 | -0.943 | 0.124 | 0.733 | 0.906 | Not significant |
| **SLC3A2** | Inf | 0.333 | 1 | 11.718 | 0.777 | 0.405 | 0.832 | 0.870 | Not significant |
| **CR1** | Inf | 0.515 | 1 | 12.721 | -0.642 | 0.291 | 0.818 | 0.824 | Not significant |
| **NECTIN1** | 0.000 | 1.000 | 1 | 11.877 | -1.252 | 0.176 | 0.733 | 0.754 | Not significant |
| **FLT1** | 0.000 | 1.000 | 1 | 11.811 | -1.153 | 0.199 | 0.733 | 0.701 | Not significant |
| **JAM3** | Inf | 1.000 | 1 | 15.430 | -0.891 | 0.249 | 0.734 | 0.604 | Not significant |
| **SLC12A2** | 0.230 | 0.545 | 1 | 12.079 | -0.479 | 0.477 | 0.832 | 0.585 | Not significant |
| **FGFR1** | 0.365 | 0.576 | 1 | 11.882 | -0.338 | 0.636 | 0.832 | 0.436 | Not significant |
| **ITGA2** | Inf | 0.515 | 1 | 15.662 | -0.301 | 0.714 | 0.864 | 0.434 | Not significant |
| **CLDN5** | 1.596 | 1.000 | 1 | 13.369 | -0.818 | 0.387 | 0.832 | 0.412 | Not significant |
| **LY75** | 0.000 | 1.000 | 1 | 11.499 | -0.679 | 0.415 | 0.832 | 0.382 | Not significant |
| **TIE1** | 0.000 | 0.491 | 1 | 11.109 | -0.097 | 0.902 | 0.927 | 0.354 | Not significant |
| **PLXND1** | 0.000 | 1.000 | 1 | 11.472 | -0.639 | 0.444 | 0.832 | 0.353 | Not significant |
| **LDLR** | 2.713 | 0.547 | 1 | 12.580 | -0.179 | 0.839 | 0.927 | 0.338 | Not significant |
| **AXL** | 0.000 | 1.000 | 1 | 11.421 | -0.562 | 0.499 | 0.832 | 0.302 | Not significant |
| **CD59** | 1.717 | 1.000 | 1 | 12.456 | 0.347 | 0.513 | 0.832 | 0.290 | Not significant |
| **CD151** | 1.000 | 1.000 | 1 | 11.998 | 0.323 | 0.664 | 0.833 | 0.178 | Not significant |
| **ITGB2** | Inf | 1.000 | 1 | 14.940 | 0.257 | 0.782 | 0.919 | 0.107 | Not significant |
| **GPC4** | 0.000 | 1.000 | 1 | 11.192 | -0.220 | 0.794 | 0.919 | 0.100 | Not significant |
| **ICAM3** | Inf | 1.000 | 1 | 15.396 | -0.117 | 0.844 | 0.927 | 0.074 | Not significant |
| **VLDLR** | 0.000 | 1.000 | 1 | 11.103 | -0.088 | 0.918 | 0.927 | 0.037 | Not significant |
| **ITGAL** | 1.000 | 1.000 | 1 | 13.166 | -0.060 | 0.927 | 0.927 | 0.033 | Not significant |
| **ADAM10** | NA | NA | NA | 16.007 | -0.962 | 0.169 | 0.733 | NA | Not significant |
| **NRP1** | NA | NA | NA | 13.271 | -0.553 | 0.315 | 0.832 | NA | Not significant |
| **ITGB3** | NA | NA | NA | 19.412 | -0.534 | 0.503 | 0.832 | NA | Not significant |
| **ICAM1** | NA | NA | NA | 13.983 | -0.589 | 0.241 | 0.734 | NA | Not significant |
| **ITGB1** | NA | NA | NA | 17.283 | -0.366 | 0.608 | 0.832 | NA | Not significant |
| **ITGA2B** | NA | NA | NA | 20.828 | -0.846 | 0.227 | 0.734 | NA | Not significant |
| **TFPI** | NA | NA | NA | 15.581 | -0.831 | 0.161 | 0.733 | NA | Not significant |
| **SLC2A1** | NA | NA | NA | 17.637 | 0.893 | 0.247 | 0.734 | NA | Not significant |
| **SLC2A3** | NA | NA | NA | 17.355 | -0.472 | 0.522 | 0.832 | NA | Not significant |
| **ITGAM** | NA | NA | NA | 15.107 | -0.104 | 0.883 | 0.927 | NA | Not significant |
| **ICAM2** | NA | NA | NA | 15.420 | -0.677 | 0.177 | 0.733 | NA | Not significant |
| **PVR** | NA | NA | NA | 13.840 | -0.352 | 0.497 | 0.832 | NA | Not significant |
| **PECAM1** | NA | NA | NA | 18.214 | -1.043 | 0.171 | 0.733 | NA | Not significant |
| **CD36** | NA | NA | NA | 16.308 | 0.310 | 0.578 | 0.832 | NA | Not significant |
| **CDH2** | NA | NA | NA | 11.319 | -0.413 | 0.649 | 0.832 | NA | Not significant |
| **VCAM1** | NA | NA | NA | 14.933 | -0.306 | 0.496 | 0.832 | NA | Not significant |
| **FGFR2** | NA | NA | NA | 11.319 | -0.413 | 0.649 | 0.832 | NA | Not significant |
| **CD9** | NA | NA | NA | 16.606 | 0.120 | 0.920 | 0.927 | NA | Not significant |
| **ITGA6** | NA | NA | NA | 17.000 | -0.289 | 0.718 | 0.864 | NA | Not significant |
| **CDH5** | NA | NA | NA | 14.728 | -0.476 | 0.340 | 0.832 | NA | Not significant |
| **GPC1** | NA | NA | NA | 11.319 | -0.413 | 0.649 | 0.832 | NA | Not significant |
| **CD81** | NA | NA | NA | 11.319 | -0.413 | 0.649 | 0.832 | NA | Not significant |
| **ADAM17** | NA | NA | NA | 11.319 | -0.413 | 0.649 | 0.832 | NA | Not significant |
| **PTPRJ** | NA | NA | NA | 16.440 | -0.459 | 0.546 | 0.832 | NA | Not significant |
| **CLEC14A** | NA | NA | NA | 11.319 | -0.413 | 0.649 | 0.832 | NA | Not significant |
| **STAB1** | NA | NA | NA | 11.319 | -0.413 | 0.649 | 0.832 | NA | Not significant |
| **LYVE1** | NA | NA | NA | 14.194 | -0.049 | 0.924 | 0.927 | NA | Not significant |

### Figure S1: MRI Brain with diffuse endothelial gadolinium leakage in a patient with Susac Syndrome

### Axial and sagittal T2-FLAIR images (A, B), post-gadolinium T2-FLAIR images (C, D), and post-gadolinium T1-weighted images (E, F) are shown. A central callosal lesion is indicated by the white arrow. Additional parenchymal lesions demonstrate a punched-out appearance on post-gadolinium T1-weighted imaging. Post-gadolinium T2-FLAIR images demonstrate nodular leptomeningeal and perivascular enhancement involving cerebrospinal fluid–filled Virchow–Robin spaces, indicated by the circle. FLAIR = fluid-attenuated inversion recovery; MRI = magnetic resonance imaging.
